# Experimental Investigation and Optimal Prediction of Maximum Forming Angle and Surface Roughness of an Al/SUS Bimetal Sheet in an Incremental Forming Process Using Machine Learning

**DOI:** 10.3390/ma12244150

**Published:** 2019-12-11

**Authors:** Raneen Abd Ali, Wenliang Chen, M.S.H. Al-Furjan, Xia Jin, Ziyu Wang

**Affiliations:** 1 College of Mechanical and Electrical Engineering, Nanjing University of Aeronautics and Astronautics, Nanjing 210016, China; engraneen@nuaa.edu.cn (R.A.A.); cwlme@nuaa.edu.cn (W.C.); meejxia@nuaa.edu.cn (X.J.); 281050988@163.com (Z.W.); 2 School of Mechanical Engineering, Hangzhou Dianzi University, Hangzhou 310018, China; 3 The State Key Laboratory of Silicon Materials, Zhejiang University, Hangzhou 310027, China

**Keywords:** incremental forming, maximum forming angle, surface roughness, microstructure, gradient boosting, Al/SUS bimetal sheet

## Abstract

Bimetal sheets have superior properties as they combine different materials with different characteristics. Producing bimetal parts using a single-point incremental forming process (SPIF) has increased recently with the development of industrial requirements. Such types of sheets have multiple functions that are not applicable in the case of monolithic sheets. In this study, the correlation between the operating variables, the maximum forming angle, and the surface roughness is established based on the ensemble learning using gradient boosting regression tree (GBRT). In order to obtain the dataset for the machine learning, a series of experiments with continuous variable angle pyramid shape were carried out based on D-Optimal design. This design is created based on numerical variables (i.e., tool diameter, step size, and feed rate) and categorical variable (i.e., layer arrangement). The grid search cross-validation (CV) method was used to determine the optimum GBRT parameters prior to model training. After the parameter tuning and model selection, the model with a better generalization performance is obtained. The reliability of the predictive models is confirmed by the testing samples. Furthermore, the microstructure of the aluminum/stainless steel (Al/SUS) bimetal sheet is analyzed under different levels of operating parameters and layer arrangements. The microstructure results reveal that severe cracks are attained in the case of a small tool diameter while a clear refinement is observed when a high tool diameter value with small step down is used for both Al and SUS layers.

## 1. Introduction

Single-point incremental forming (SPIF) is a die-less sheet metal forming process where the hemispherical tool forms the sheet locally with incremental plastic deformation. This tool follows the designed tool path in layers to produce the desired parts. The SPIF process has higher formability, and lower forming force and cost compared with the conventional forming processes which makes it suitable for metal forming sheets. Generally, this process forms single metal sheets; however, recently the potential has increased to form multi-layer sheets. The multi-layer sheets are increasingly used in automotive and aerospace industries because of their ability to perform multiple functions. These sheets have good mechanical, physical, and chemical properties that are not offered in the single layer sheet, making them superior in different industrial applications. As the composite sheets combine different materials that have different characteristics, this strengthens the conductivity, corrosion resistance, and the yield strength of the combined sheet. The application of such processes to produce the desired composite parts combines the characteristics of both the forming process and the parent materials to enhance the quality and the formability of the low formable materials and reduce the cost and the weight of manufactured parts.

The SPIF of composite sheets has generated considerable recent research interest. In the study of Al-Ghamdi and Hussain [1], the formability improvement of a Cu/steel bimetal sheet during SPIF was better than that in the stamping process due to the limitation of the delamination in the former process. In their further work [2], the relation between the maximum forming angle and the process parameters of the tri-layer Cu/steel/Cu was studied experimentally. The results of the study revealed that a complex relation was observed between the operating parameters of the tri-layer sheet. Gheysarian and Honarpisheh [3] investigated the effect of the layer arrangement, tool path, and tool diameter on formability (i.e., fracture depth) and surface quality of Al/Cu bimetal sheets. The results showed the improvement in the formability corresponding to a large tool diameter, spiral tool path, and copper layer as a contacted sheet. Later, Honarpisheh et al. [4] focused on the improvement of the maximum forming angle of Al/Cu bimetal sheets through multi-objective optimization in the SPIF process. Recently, Liu and Li [5] studied the effect of some operating factors on the formability and surface roughness of Al/Cu bimetal sheets.

In addition to the formability, the surface quality of the SPIF parts represents an important aspect from the service life and aesthetic view of the desired parts. Usually, this quality is defined with the average roughness value (Ra) and, to meet the requirements of the customers, the surface roughness of the produced desired parts should be within an acceptable range. The average surface roughness (Ra) is the most effective parameter that can be used to evaluate the quality of incrementally-formed parts since it gives information on the behavior of the contact and non-contact surfaces with the forming tool [6]. The main parameters that affect the contact surface roughness of a single-layer sheet are the step size, tool diameter, feed rate, and rotational speed [7]. Furthermore, the non-contact surface roughness is highly sensitive to the ratio of the forming angle to the step size, which is called the shape factor [8]. Numerous studies have been focused on the surface roughness of the single-layer sheets with respect to the operating parameters [7,9,10,11,12]. Nevertheless, little attention has been given to the surface roughness in the bilayer SPIF. For example, the recent study of Wei et al. [13] investigated the surface roughness of single-layer sheets of Al1060 and bilayer sheets of Cu/Steel. Furthermore, the recent work of Al-Ghamdi and Hussain [6] analyzed the contact and non-contact surface roughness of tri-layer (Cu/Steel/Cu) sheets; however, the internal and external layers have the same material type. Therefore, studying the effect of the layer arrangement on the surface roughness of bilayer sheets that have different materials still need an investigation to understand, first, how the different levels of process parameters affect the surface quality of the internal and external layers of bilayer sheets and, second, whether the layer arrangement plays a major rule in roughening the bilayer sheets or not.

More recently, many studies in the SPIF of monolithic sheets have been focused on establishing the predictive models based on different approaches. These approaches include deep learning, statistical, and analytical methods. Kurra et al. [14] predicted the average and maximum surface roughness of single steel sheets using both artificial neural network (ANN) and support vector regression, taking into consideration the effect of tool diameter, step size, feed rate, wall angle, and lubrication type. In addition, a predictive model was established based on an ANN for the formability and surface roughness of an AA5052-H32 single sheet. The best network was determined based on the number of neurons and the transfer function in the hidden layer [15]. Maji and Kumar [11] developed forward and inverse models to predict the forming angle, sheet thickness, and surface roughness using an adaptive neuro-fuzzy model and response surface methodology (RSM). Basak et al. [16] predicted the maximum forming angle and the quality of the incremental formed parts using RSM (Box–Behnken design). Yao et al. [17] established the relation between the process variables (step size, tool diameter, wall angle, and sheet thickness) and surface quality using the response surface methodology. An attempt was made to improve the formability and the quality of aluminum parts by Baruah et al. [18] using grey relation analysis based on the analysis of variance (ANOVA) technique. Prediction the formability and surface roughness using the analytical models may not give precise information due to the complexity of the plastic deformation induced in the current process where bending, shearing, and stretching deformations are shared in this process. In order to build an analytical model it is difficult to share all of these deformations; for simplicity some of them should be ignored. Ai et al. [19] built an analytical model to analyze the deformation stability in the SPIF process. In this model, they focused on the plastic deformation induced in the contact and non-contact area in order to enhance the formability of the formed parts. They considered the plastic deformation resulting from the stretching and bending effects, whereas the shearing effect was neglected for the purpose of simplicity. Durant et al. [20] derived an analytical model for predicting the surface roughness of pyramid part and compared it with the experimental one. Recently, Chang and Chen [21] developed an analytical model to estimate the surface roughness taking into consideration the elastic deflection, plastic deformation, and non-uniform thickness distribution. This model is more general than the predictive one of Durant et al. [20].

Based on the literature review, artificial intelligence is widely used to develop predictive models in SPIF. Using machine learning methods can help to reduce the number of experiments required for studying the relationship between the formability and the quality of bimetal sheets under different layer arrangements. The machine learning methods are strategies inspired by nature that are able to establish predictive models that can be used in different applications. The application of these methods to model the SPIF of bimetal sheets is an open issue. Using finite element method (FEM) to simulate the SPIF process is computationally expensive, and different approaches that minimize the simulation trials are required. Nowadays, machine-learning techniques are used widely in different industrial applications and have proven their effectiveness to predict different complex problems. However, few studies have focused on the formability and, in particular, on the surface quality of bimetal sheets; however, no attempt has been made for predicting the formability and surface roughness in the bimetal sheets. Moreover, among the different bimetal sheets, the surface roughness of Al/SUS bimetal sheets has not been investigated yet. Due to the different applications of the used material, and considering the surface quality as one of the weak points in the current process, more attention should be given to the surface roughness of bimetal sheets. Especially, the quality of the SPIF parts is highly limited by the step size factor and reducing this factor makes the process time very long, hence, reducing the efficiency of the SPIF process. Therefore, creating a predictive model can help to reduce the number of the experiments and reducing the forming time by predicting the surface roughness of the bimetal sheets. Recently, different machine learning techniques have been used in the prediction and optimization of different industrial applications and using the gradient boosting regression tree (GBRT) as one of the machine learning methods due to the good performance of this method in different nonlinear problems can help to establish good models to improve the quality and the formability of the composite formed parts. However, according to the authors’ knowledge, no such work has been implemented in the literature using the previous method. Furthermore, the effect of layer arrangement and different combinations of process parameters on the microstructure of the aluminum/stainless steel (Al/SUS) bimetal sheet through SPIF has not been investigated yet. 

In this study, the GBRT is applied to establish the correlation between the operating parameters and the formability and the quality of Al/SUS bimetal sheets under different layer arrangements. The GBRT is employed as a machine learning technique to achieve an accurate and rapid prediction model. First, the D-Optimal design based on RSM is adopted to establish the training dataset due to good features of this design, which meet the requirements of the current work. This design is implemented using a truncated pyramidal part with variable wall angles through the SPIF process. Then, the tuning parameters of the GBRT are carried out using the grid search cross-validation (CV) method. The optimal prediction model is established according to the optimum GBRT parameters. Finally, the microstructure observation for the contact and non-contact surfaces is analyzed based on different operating parameters and layer arrangements of truncated pyramid parts.

## 2. Experimental Procedures

The design of experiment (DOE) technique was chosen to investigate the maximum forming angle and the surface roughness of Al1050/SUS304 bimetal sheets in the SPIF process. The experimental plan was developed based on RSM using Design Expert DX10 software (Stat-Ease, Minneapolis, MN, USA). RSM is an effective tool of DOE, which uses a series of designed experiments to acquire an optimal response. It is a group of statistical techniques for fitting empirical models with a set of experimental data. These data are gained from the relationship between dependent variables (responses) and independent decision variables (inputs) to minimize and maximize the characteristics of the response [22]. In the current study, the quadratic model is adopted to determine the desired response and the mathematical model can be expressed as follows [23]:

(1) $$Y=b_{0}+\sum_{i=1}^{k}b_{i}x_{i}+\sum_{i=1}^{k}b_{ii}x_{i}^{2}+\sum_{i=1}^{k}\sum_{j\geq k}^{k}b_{ij}x_{i}x_{j}+e$$

where $b_{0}$ is the constant term, $b_{i}$, $b_{ii}$, and $b_{ij}$ represent the linear, quadratic, and interaction coefficients, respectively, and e is the error corresponding to the response y. D-Optimal design is a more flexible design by sensing the irregularity of the experimental region and trying to span the space as well as possible. This design can be developed with quantitative factors (continuous or limited to specific levels) and qualitative (categorical) factors. The D-Optimal design is a computer-created design that consists of the best subset of tests that are selected from the so-called candidate set. The candidate set is the tool of theoretical possible and practically conceivable experiments that could be carried out. Moreover, the D-Optimal design reduces the number of experiments compared with the other response surface methods (i.e., Box–Behnken design and central composite design) [22]. According to these reasons, this design was selected to meet the requirements of the current investigation.

**Figure 1:**
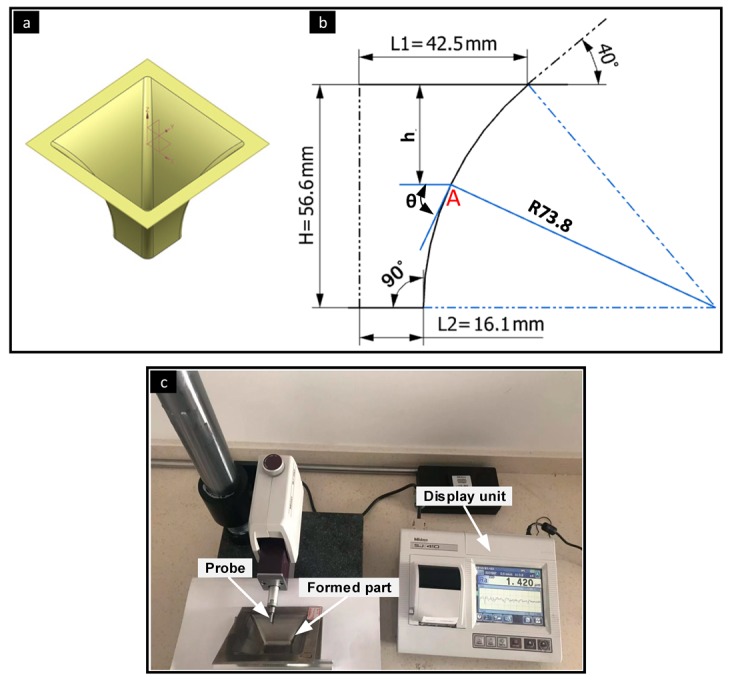
Experimental configuration. (**a**) Test geometry; (**b**) geometric illustration of pyramidal part; (**c**) surface roughness measurement.

Four parameters were selected as input factors for the adopted design. Three numerical factors with one categorical factor were varied over three levels and two categories. These factors consist of tool diameter (d), step size (∆z), feed rate (f), and layer arrangement (LA) as shown in Table 1. In this table, the low level of the feed rate was selected as 1000 mm/min because using a feed rate lower than this value makes the process time very long and reduces the efficiency of the current process. The high level of this parameter was selected as 3000 mm/min based on the machine specifications. In order to distinguish the stacking of the layers, two expressions are used. The expression of SUS/Al denotes to stainless steel layer contacts the tool and the aluminum layer contacts the backing plate. The maximum forming angle (θ) and the surface roughness (Ra) were considered as output factors. The experimental plan was built with 22 runs based on D-Optimal design as shown in Table 2. The input parameters were used as inputs for the GBRT model to build a good model that is able to predict the maximum forming angle and the surface roughness, which represents the outputs for the former model. The formability of the bimetal sheet was evaluated using the maximum forming angle (θ) method for the monolithic sheet as described in Hussain and Gao [24]. For this purpose, a variable wall angle frustum pyramid with the base of 85 mm × 85 mm was used as depicted in Figure 1b. The pyramidal part, with continuously variable angles from 40° to 90°, was formed until fracture. In this figure, H and h represent the designed depth and the expected final depth at the fracture point A, respectively. At this point the θ represents the maximum forming angle.

The SUS/Al bimetal sheet has superior characteristics, including good formability, corrosion resistance, electrical conductivity, and heat induction [25]. Additionally, this bimetal sheet has different applications in aircraft, and electrical industries [26]. Therefore, this bimetal sheet was used in the current investigation with total thickness of 2 mm. The hot rolling process was used to produce such a bimetal sheet with 1.5 mm and 0.5 mm thicknesses of Al1050 and SUS304, respectively. Further details of the mechanical properties of the materials used in the current investigation can be retrieved from the previous work of the authors [27]. A three-axis CNC milling machine with a free rotational hemispherical forming tool was used to perform the SPIF process. Mineral oil was used in the contact area between the inner surface of the bimetal sheet and the forming tool to minimize the friction effect and improve the surface quality of the final part [12]. Z-level tool path was created in NX 10 software (Siemens PLM, Berlin, Germany) to produce the pyramidal parts according to the designed plan. Moreover, the sheets were cut into 130 mm × 130 mm and clamped along its edges using the blank holder and the backing plate. 

In order to evaluate the quality of the produced bimetal parts, the average surface roughness (Ra) was measured using a Mitutoyo SJ-410 (Kawasaki, Japan) roughness tester as shown in Figure 1c. The Ra value of each pyramidal part was measured at the same depth five times and the average value was calculated for easy comparison and more accurate results. The Ra was recorded perpendicular to the forming tool movement with cut of length 0.8 mm and sampling length 6 mm. In order to test the microstructure changes in the contact and non-contact surfaces for both layer arrangements, small samples were cut using an electric discharge machine (EDM, Dk7750, Suzhou Uslugi Machine Co., Ltd., Suzhou, China). For easy comparison, all samples were cut at the same location and tested using an RH-2000 digital microscope (Hirox Europe Ltd., Hackensack, NJ, USA) with 10 mm × 10 mm sizes.

## 3. Development of the Machine Learning Model

### 3.1. Proposed Methodology

**Figure 2:**
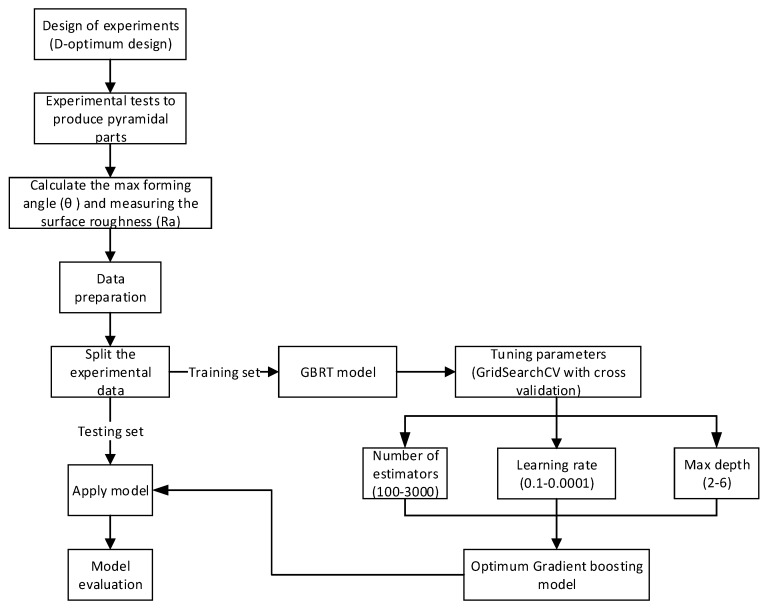
The proposed methodology for modeling and determining the optimum predictive model.

The methodology of determining the optimum GBRT model for the current study is presented in Figure 2. Three numeric parameters (d, ∆z, f) and one categorical parameter (LA) are selected as inputs for the predictive model while θ and Ra are selected as outputs. A python program was written according to the flow chart shown in Figure 2. In order to quantify the correlation between input parameters and the maximum forming angle and surface roughness of the bimetal sheets, it is of significant importance to study the contribution of each input parameter to the outputs. From Figure 2 it can be seen that the first step is to collect the experimental data by creating a robust design that can help to create a good predictive model. In this step, a pyramidal part with continuously variable wall angles was selected to perform the experimental plan. Then, the outputs were determined according to details described in the previous section. The second step is determining the optimum parameters of GBRT for each predictive model. The optimum parameters were evaluated based on the grid search CV method. This method is combined with the cross-validation method where the latter is an effective method for small data analysis, which is used to avoid the noise problems caused by small samples. The most important parameters that influence the results of the predictive model are number of trees, learning rate, and the maximum depth of the trees. Therefore, these parameters were tuned to determine the optimum GBRT models. In the last step, it is necessary to evaluate the accuracy of the final optimum models using new data, which are not included in the training stage. More details will be described in the following section.

### 3.2. Gradient Boosting Tree

The gradient boosting regression tree (GBRT) is one of the most popular machine learning algorithms, which can be used to predict the continuous values and classify samples into different categories based on learning a sequential dataset. In this algorithm, the weak learners are learned sequentially and combined together to create a strong predictive model. Unlike other machine learning algorithms, the GBRT algorithm starts by making a single leaf, instead of a tree or stump (a very short tree). This leaf represents an initial guess for the outputs for all of the samples which represents the average value of the desired output. Then, the GBRT builds a tree based on the residuals made by the previous tree and scales the tree’s contribution to the final prediction with a learning rate (Lr). These residuals represent the difference between the observed values and the predicted values, which is called the pseudo residual. The GBRT continues to build trees in this fashion until reaching the maximum specified number of trees or adding additional trees no longer significantly reduces the size of the residuals. The initial predicted value that minimizes the loss function can be described as follows:

(2) $$F_{0}(x)=\underset{F}{\mathrm{argmin}}\sum_{i=1}^{n}L(y_{i},F(x))$$

where *n* is the number of input variables, $y_{i}$ is the observed values, F(x) is the predicted value, and L is the loss function. By solving the above equation it can be noticed that the initial predicted value $F_{0}\left(x\right)$ represents the average of the observed values, which is just a leaf as mentioned earlier. The residual for each sample is a sensitive term which represents the deviation of the loss function with respect to the predicted value. In order to optimize the GBRT by the steepest descent method, the negative gradient $r_{im}\left(x_{i}\right)$ can be determined using the following equation:

(3) $$r_{im}(x_{i})=-\frac{\partial L(y_{i},F_{m-1}(x_{i}))}{\partial F_{m-1}(x_{i})}$$

where *r* is the residual, *m* is the number of tree $x_{i}$ is the input values, and i is the number of samples. By modeling the pseudo residual, the weighting factor $\gamma_{m}$ that can be used to build next tree $h_{m}\left(x\right)$ is given by:

(4) $$\gamma_{m}=\underset{\gamma }{\mathrm{argmin}}\sum_{i=1}^{n}L(y_{i},F_{m-1}(x_{i})+\gamma h_{m}(x_{i}))$$

where $h_{m}\left(x_{i}\right)$ represents weak learner which trained based on the residuals. Finally, after making a new prediction for each sample based on the previous learners, the predicted strong learner value $F_{m}\left(x\right)$ can be defined as follows:

(5) $$F_{m}(x)=F_{m-1}(x)+\gamma_{m}h_{m}(x)$$

where $F_{m-1}\left(x\right)$ represents the predicted value from the previous model and $\gamma_{m}h_{m}\left(x\right)$ represents the weighted current model. Recently, GBRT has become more popular to predict the complex problems as well as proved its effectiveness in different industrial applications [28]. This is because the GBRT combines many weak learner models that increase the ability to produce an effective predictive model. In the GBRT, adding more trees to the model may raise the risk of overfitting, which is a common issue in the different machine learning methods. To improve the generalization of the predictive model, tuning the GBRT parameters is inevitable. These parameters include boosting hyperparameters which represent the number of trees (M) and the learning rate (Lr), and the main tree hyperparameter, which includes the size of the tree (H). To solve the overfitting problem, the optimum number of trees should be determined based on the minimum loss function, which can help with the better generalization of the predictive model. The learning rate is another important parameter that controls the influence of the weight of each tree in the resulting model. In general, the value of this parameter ranges from 0 to 1. Setting a small value for the learning rate increases the accuracy of the predictive model. On the other hand, this results in an increase in the number of trees required to be added to the model. In addition to the number of trees, setting the suitable value of the learning rate can contribute to overcoming the overfitting problem as well. In the GBRT, the size of the trees is also called the tree depth and can be controlled for more robust models. The small size of trees describes little information of the problem which results in poor performance and the large size of trees describes too much information about the problem, leading to overfitting the model and reducing the ability to predict the new dataset. In order to evaluate the performance of the predictive models accurately, two criteria are selected, root mean squared error (RMSE) and mean absolute percent error (MAPE), as described in Equations (6) and (7). The RMSE is the square root of the average of the quadratic deviations between the predicted value and the actual value.

(6) $$RMSE=\sqrt{\frac{1}{k}\sum_{i=1}^{k}{\left(y-\bar{y}\right)}^{2}}$$

(7) $$MAPE=\frac{1}{k}\sum_{i=1}^{k}\frac{\left|y-\bar{y}\right|}{y}\times 100\%$$

where *k* is the size of samples, *y* is the actual value, and $\bar{y}$ is the corresponding predicted value.

## 4. Results and Discussion

In this section, the analysis of the maximum forming angle and the quality of the Al/SUS bimetal sheet with respect to the process parameters are performed. Then, the microstructural changes of the contact and non-contact surfaces are represented with different levels of operating parameters and layer arrangements. Furthermore, the optimum GBRT parameters are determined based on the grid search CV method. Finally, the prediction results of the GBRT models are evaluated and discussed.

### 4.1. Analysis of Experimental Results

#### 4.1.1. Feature Importance Based on GBRT

**Figure 3:**
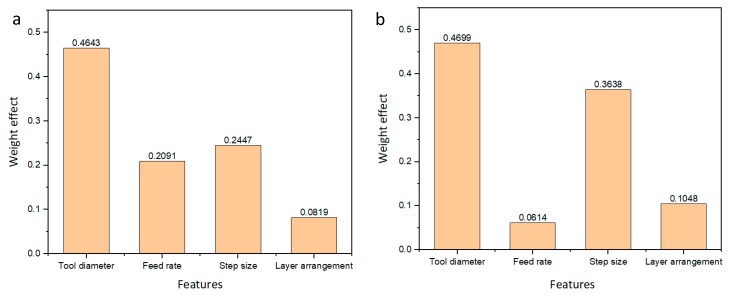
The importance of each feature on the (**a**) max forming angle, and (**b**) surface roughness.

Features represent the input parameters which are used in the training and testing of the GBRT model. From the mathematical view, the features of the problem are the independent variables that are used to solve the equation. To show the importance of each feature on the responses, the machine learning techniques can also be used. Usually, to determine the effect of each factor, the ANOVA method can be used for this purpose. However, the application of GBRT as a machine learning technique can be very helpful to achieve such a purpose. The weight effect means the contribution of each input parameter on the response, in other words, how much each factor decreases or increases the maximum forming angle and the surface roughness. The high value of this weight means that the factor corresponding to this weight has the greatest influence on the changes in that response. As can be seen from Figure 3a, the most important parameters that affect the maximum forming angle are forming tool followed by the step size. These results are consistent with the work of Honarpisheh et al. [4]. In Figure 3a, although the layer arrangement has less influence on the maximum forming angle compared with the other operating parameters this effect is still significant and cannot be removed from the current analysis. Moreover, in Figure 3b the importance of the features on the surface roughness showed that the tool diameter also has the highest effect while the feed rate has the lowest effect. This result is consistent with the previous work of Echrif et al. [7]. Nevertheless, the feed rate has non-negligible effect on surface roughness. Considering the effect of layer arrangement on surface roughness, this analysis confirms that the material type can affect the surface roughness as mentioned previously in the single layer sheet [13]. Moreover, the layer arrangement has a greater effect on the surface roughness than the maximum forming angle of the bimetal incremental forming sheet. As a result, all features have a considerable effect on the responses.

#### 4.1.2. Experimental Parameters Effect

**Figure 4:**
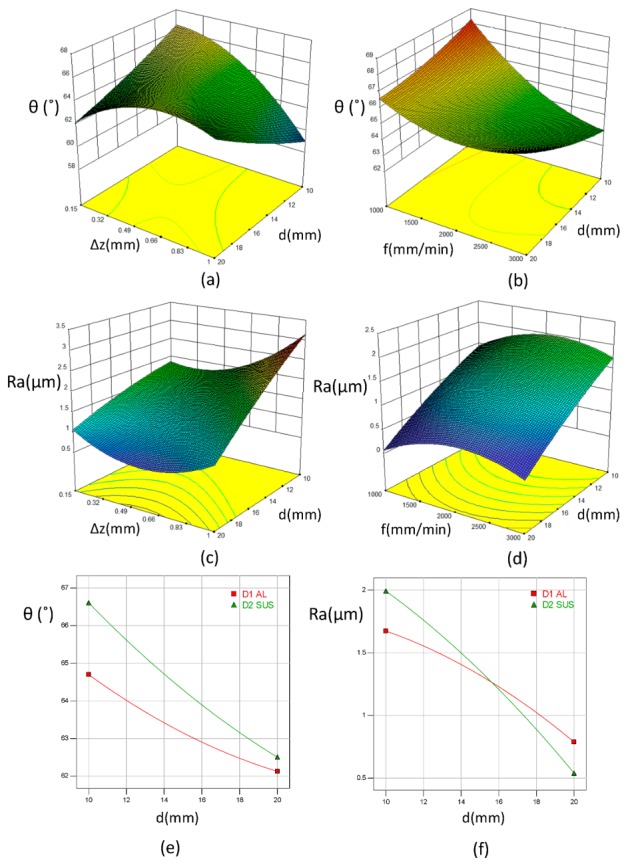
Response surface and main effects plots for different levels of the operating parameters. (**a**); θ with **∆z** and d (**b**) θ with **f** and d; (**c**) Ra with **∆z** and d; (**d**) Ra with **f** and d; (**e**) θ with d and LA; (**f**) Ra with d and LA.

Figure 4a–f represents the correlation between the operating parameters and the responses (i.e., the maximum forming angle and surface roughness) with layer arrangements. In order to study the effect of two parameters with the responses, the other parameters are kept constant at the average values. Figure 4a,b demonstrates that a lower step size and feed rate lead to reduce the maximum forming angle of the bimetal parts with an increase in the tool diameter. The same observations are reported in the previous study of Basak et al. [16]. However, the effect of step size is more obvious than the feed rate effect, which is consistent with the feature importance observations in the previous section. Moreover, under the average levels of the process parameters, the maximum forming angle decreases as the tool diameter increases for both layer arrangements (see Figure 4e). For more details about the maximum forming angle behavior of this bimetal sheet can be found in the previous work of the authors [27].

The surface roughness of the incrementally-formed bimetal sheets plays an important characteristic in the composite sheet metal forming, particularly with increasing the demand to produce the composite parts in various manufacturing processes. Therefore, studying the effect of the operating parameters can help in improving the quality of the bimetal formed parts. From Figure 4c, it can be seen that the surface roughness decreases as the tool diameter increases. However, the increase in the step size is mutually opposite to the tool diameter trend, where the increase in the step size causes a significant increase in the surface roughness. In fact, the effect of tool diameter and step size on the surface roughness of the bimetal sheet has the same trend as in the monolithic sheet [9]. The increase in the tool diameter showed an improvement in the quality of the formed parts due to increase the contact area between the forming tool and the internal layer which results in reducing the stresses [29]. In addition, reducing the step size has a positive effect on the surface roughness. As the step size decreases the scallop height, which represents the height between two adjacent tool paths, decreases the waviness that leads to decrease surface roughness [7,9,11]. In Figure 4d, the interaction between the tool diameter and the feed rate is represented and the surface roughness corresponding to different feed rates is depicted. As can be seen from Figure 4f, a clear interaction between the tool diameter and the layer arrangement is observed where, at a small tool diameter, the surface roughness in the SUS/Al bimetal sheet is higher than that in the Al/SUS bimetal sheet. However, this behavior is mutually opposite in the case of a large tool diameter. The results in the case of a small tool diameter are consistent with the results obtained in the previous study of Radu and Cristea [9] for monolithic sheets. According to the study of Radu and Cristea [9], the surface roughness of Al1050 monolithic sheet was better than that of SUS304 monolithic sheet. However, due to the small range of operating parameters used in the study of Radu and Cristea [9], where the largest tool diameter was 10 mm, no interaction was observed between the material type and tool diameter. More details will be discussed in the Section 4.1.3. Furthermore, compared with Ra values for monolithic sheets in Radu and Cristea [9], the roughness in single Al and SUS sheets are less than that in Al/SUS bimetal sheets (i.e., in the current study) which may indicate that the difference in the materials’ strength of the bimetal sheets has an effect in increasing the surface roughness of latter sheets as confirmed in the recent study of Al-Ghamdi and Hussain [6].

As a result, there is a contradiction between the maximum forming angle and the surface roughness of bimetal sheets. At a small tool diameter, although the SUS/Al bimetal sheet has a higher maximum forming angle compared with the Al/SUS bimetal sheet, the surface roughness has the opposite trend in this condition. Therefore, a proper selection of the operating parameters and layer arrangement is a vital issue to find a balance between these two aspects.

#### 4.1.3. Microstructure Analysis of the Bimetal Surfaces

**Figure 5:**
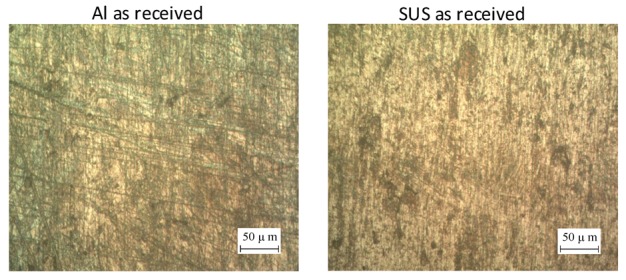

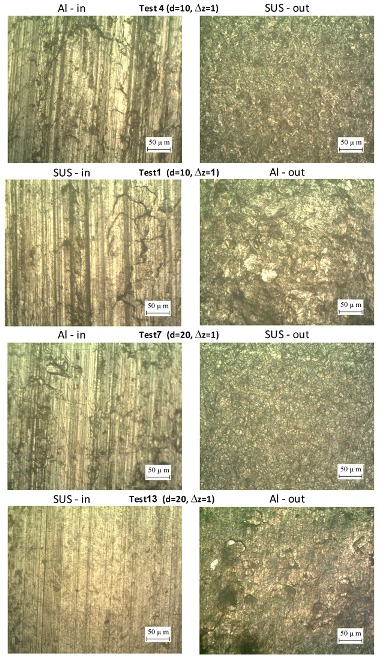

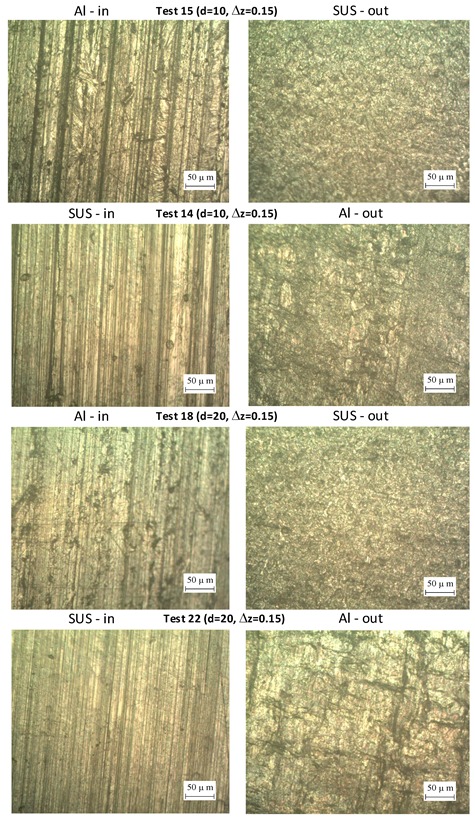
The microstructure of the contact and no-contact surfaces for Al/SUS and SUS/Al layer arrangements.

In this section, the effect of different combinations of the process parameters on the microstructure of the contact and non-contact surfaces is shown in Figure 5. In this figure, the first two samples show the microstructure of unformed Al and SUS sheets and the other samples show the microstructure of the formed parts under different layer arrangements. Above each sample a number was given which corresponds to the number of the test in Table 2. The contact surfaces in Al/SUS and SUS/Al bimetal sheets show the worst quality using a small forming tool (i.e., 10 mm) and large step size (i.e., 1 mm), especially when the SUS layer contacts the tool. These observations are consistent with the obtained results in the previous section (see Figure 4f). Under the small tool and large step both contact surfaces suffer from severe micro cracks (see Figure 5, test 1 and test 4). In addition, with a small tool diameter both surfaces experience clear striations under different levels of step size (see Figure 5, test 1, test 4, test 14 and test 15). Nevertheless, with a large tool diameter (i.e., 20 mm), a significant improvement in the microstructure is observed for both step size and layer arrangements. However, a good improvement in the SUS structure is attained when SUS represents the internal layer using a small step size and large tool diameter (see Figure 5, test 22).

Although the main focus of the current study was to analyze the contact surface roughness, the microstructure of some non-contact surfaces was also analyzed for the purpose of comparison. The non-formed Al and SUS sheets have an average roughness of 0.107 μm and 0.094 μm, respectively. These values are lower than the surface roughness of external Al and SUS layers after the SPIF process. This refers to the orange peel effect, which is very common in this process, as was observed by Jeswiet et al. [29]. Comparing different levels of operating parameters, it can be seen that the external Al and SUS surfaces experience roughening (i.e., orange peel) because of the severe plastic deformation resulting from the SPIF process. However, the roughening in the external Al layer is more obvious than that in the external SUS layer, especially with a small tool diameter. In fact, the amount of free surface roughening is affected by the amount of plastic deformation and highly depends on the size of the grains [30]. In addition to the orange peel, clear micro crack and voids are observed on the external Al layer using the large tool diameter and step size. For both step size increases the tool diameter shows a better refinement of grains (structure) in the case where SUS represents the outer layer. However, a significant decrease in the roughening is observed when SUS forms the external layer using a large tool diameter and small step size. This observation is similar to what is observed in the microstructure of the contact surfaces. From the above analysis it can be concluded that the structure refinement in the external layers is better than that in the internal layers. Moreover, using the large tool diameter and small step size with the SUS/Al layer arrangement can improve the surface quality of the bimetal parts. At the same time, using the latter layer arrangement results in high roughening as the Al layer represents the external layer; therefore, the suitable selection of the process conditions is important to control the effect of the surface roughness in the contact and non-contact layers of the desired parts.

### 4.2. Analysis of Modeling Results

#### 4.2.1. Optimum GBRT Parameters for Maximum Forming Angle and Surface Roughness Models

In the current study, the GridSearchCV method is used to perform the parameters’ tuning and determine the optimum model corresponding to each response. Tuning the hyperparameters manually one-by-one is tedious and requires more time; therefore, using the GridSearchCV method from the sklearn library can make this process easier for tuning hyperparameters. It searches over every combination of the hyperparameters’ values to evaluate which combination leads to the best performance. Among the different machine learning techniques, the cross-validation process is implemented to search for the optimum parameters which give the best accuracy levels. After finding the optimum hyperparameters, these chosen parameters are used in the GBRT models in order to predict the corresponding response (see Figure 2 and Table 3). 

Using the KFold cross-validation with five folds results in 600 combinations of parameters for each output. The values of the GBRT parameters ranging from 2–6, 100–3000, and 0.1–0.0001 for the size of the trees (H), number of the trees (M), and learning rate (Lr), respectively. Further increases in the size of the trees (i.e., the maximum depth greater than 6) leads to poor performance of the predictive model. Moreover, using the maximum depth less than 2 is possible but it is insufficient in the prediction of the responses. Therefore, only the maximum depth in the range from 2–6 is studied.

**Figure 6:**
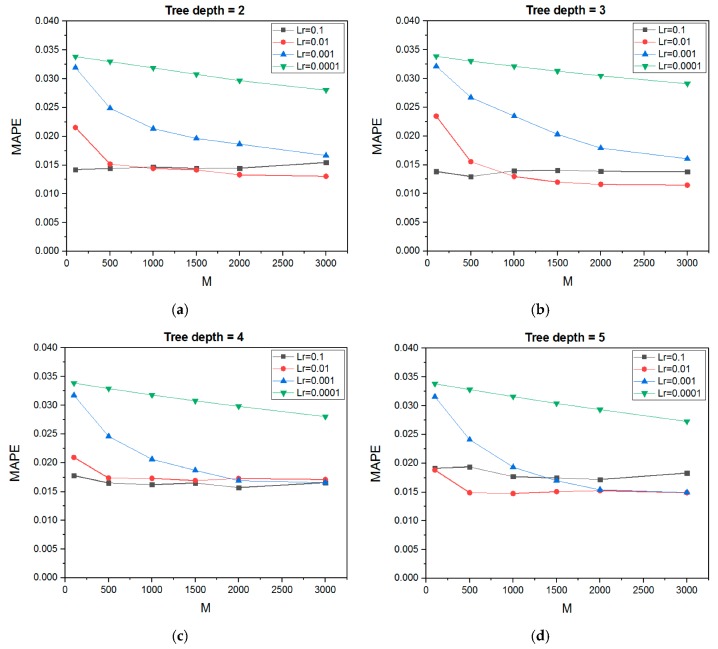
The correlation between the number of trees and learning rates with corresponding error under different trees depths. (**a**) H = 2; (**b**) H = 3; (**c**) H = 4; (**d**) H = 5.

The correlation between the average error and the parameters’ combination is shown in Figure 6. The slope of the curves varies with different values of the learning rate. As the Lr is an important parameter which contributes of each regression tree to the final predicted result. From this figure it can be seen that a very small learning rate (i.e., 0.0001) shows poor accuracy compared with the mean value for the different values of tree depth. Moreover, a high learning rate (i.e., 0.1) leads to the overfitting problem. The best contribution of the learning rate is shown at a value of 0.01 as it reduces the MAPE significantly for different trees depths. In addition, increasing the M leads to a decrease in the MAPE; however, after a certain value a further increase in M does not improve the performance of the model. This is because of the construction way of the GBRT model, adding new trees sequentially attempts to model and correct the errors made by the previous trees. Quickly, the model attains a point of decreasing the finding.

Furthermore, it is well known that increasing the size of trees leads to an improvement in the accuracy of the prediction; however, the better performance of the prediction model does not always correspond to the higher value of maximum depth. The correlation between the hyperparameters of the surface roughness model is approximately the same as that of the maximum forming angle model; therefore, only the tuning parameters for maximum forming angle model are presented in Figure 6. In fact, the results in Figure 6 of different combinations of Lr, H, and M parameters result from implementing the GridSearchCV method to determine these optimal parameters based on the relation between the inputs and the outputs used in the current study. The final results of this method are shown in Table 3 which represent the optimum combination of GBRT parameters for both the maximum forming angle and the surface roughness models based on the analyzed results in Figure 6. These optimum combinations can be defined based on the minimum MAPE. From Table 3 it can be shown that the MAPE for the surface roughness is higher than that in the maximum forming angle model. This is because using the MAPE with the small output values (i.e., Ra values) has a problem since it takes only the observed values in the denominator (see Equation (7)) which leads to a reduction in the overall accuracy [31]. Later, in the evaluation section, suitable criteria will be used for both models to show the accuracy of the predictive models.

#### 4.2.2. Prediction of Maximum Forming Angle and Surface Roughness

The gradient boosting tree model can recognize regression and classification data, such as gradient boosting regression and gradient boosting classification. As the responses are numeric continuous values, in this study the GBRT algorithm is used, which was proposed by Friedman [32]. To evaluate the performance of this algorithm, it is necessary to split the dataset into training and testing sets. First, the model is trained using training data, and then makes prediction for the trained model based on the test set, which includes the new dataset (i.e., unseen data in the training model). The predicted model should be evaluated against the expected results in order to ensure the generalization of the final model. 

**Figure 7:**
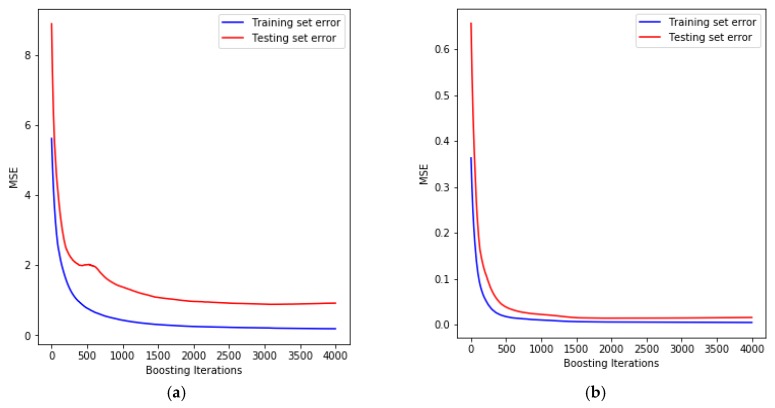
The performance of optimum GBRT model for estimating (**a**) the maximum forming angle, and (**b**) the surface roughness.

The aim of developing such model is to create a model that is able to predict the unseen data accurately. This aim can be achieved using a good statistical technique (e.g., D-Optimal design in the current study) where the training data are selected carefully to evaluate the accuracy of the optimum model using the new dataset. Using the cross-validation technique can reduce the noise generated from the small size of the training data and ensure the generalization of the optimum model to predict the new dataset. The data that were extracted from the experimental tests are listed in Table 2. As shown in Section 4.2.1, the optimum parameters were obtained for each model based on the grid search method. Within these prediction models, the original data were used without any data processing methods (e.g., normalization of the data). Based on the optimum depth of tree and learning rate, the variation of the mean squared error (MSE) during the training and testing models is shown in Figure 7. In this figure, MSE decreases as the boosting iterations (i.e., the number of trees) increases during the training and testing stages. This results in the best performance of the predictive model at 3000 and 2000 trees, having MSE values of 0.89557 and 0.01386 for the maximum forming angle and surface roughness, respectively. As can be seen from both models the training and testing data are smoothly varied with the iterations. This means the current models have higher reliability and stability to predict the maximum forming angle and surface roughness of Al/SUS bimetal sheets.

**Figure 8:**
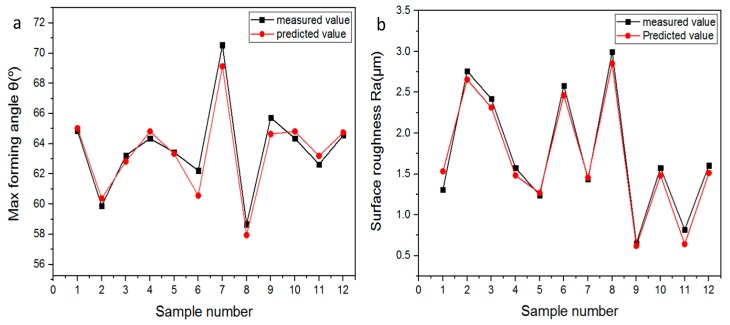
Comparison between the observed and predicted value. (**a**) Maximum forming angle; (**b**) Surface roughness.

The results of the validation tests for the optimum models are shown in Figure 8. The validation data were selected randomly to ensure the performance of the predictive model for the maximum forming angle and surface quality of the Al/SUS bimetal sheet. Twelve data points were used for validation, which includes six data points for Al/SUS bimetal sheet and the rest of the data for the SUS/Al bimetal sheet. As shown in Figure 8a, the GBRT model for predicting the maximum forming angle is closest to the experimental data with an RMSE value of 0.946346. Similarly, the prediction result of surface quality matches well with the observed one with an RMSE value of 0.117723.

**Figure 9:**
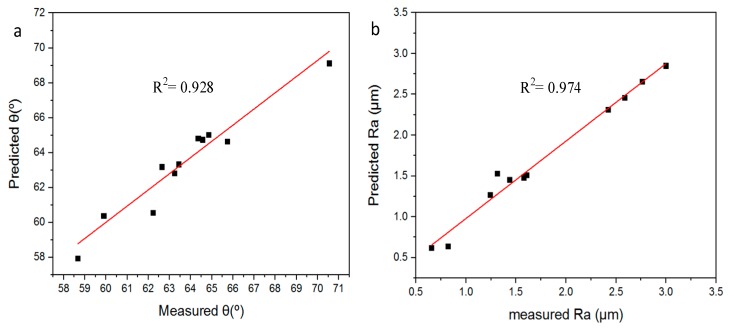
Correlation between the predicted and observed data. (**a**) Maximum forming angle; (**b**) surface quality.

Figure 9 shows the regression analyses of the optimum models. The red line represents the fitting line of the experimental and predicted data. The R-squared value of the two models shows a good correlation between the actual and the predicted data. The results of the predictive models show that the GBRT is a good algorithm to establish a high-accuracy model, which is expected to have the applicability for prediction and optimization of operating parameters to achieve a desired maximum forming angle and surface quality in the bimetal sheets.

## 5. Conclusions

In this study, predictive models were developed for predicting the maximum forming angle and surface quality based on machine learning. The correlation between the operating parameters and responses was established with the D-Optimal design under different layer arrangements. The data were generated by implementing this design to produce pyramidal bimetal parts with variable wall angles. The surface topography for Al/SUS and SUS/Al layer arrangements was analyzed under different levels of operating factors. The optimal predictive model was defined based on the grid search CV method. The gradient boosting regression tree (GBRT) was used for feature importance, model training, and verification. The main findings from the current work are summarized as follows:

1. The tool diameter has the highest effect on the maximum forming angle of the Al/SUS bimetal sheet, while the layer arrangement feature has the smallest effect, but this effect cannot be neglected. The GBRT model with a maximum tree depth of 3, trees numbering 3000, and a learning rate of 0.01 is suitable for the maximum forming angle prediction.
2. The tool diameter has the highest effect on the surface roughness of the Al/SUS bimetal sheet, while the feed rate feature has the smallest effect, but this effect cannot be neglected. The GBRT model with a maximum tree depth of 3, trees numbering 2000, and a learning rate of 0.01 is suitable for the surface roughness prediction.
3. The high value of tool diameter has a negative effect on the maximum forming angle of the SUS/Al bimetal sheet but a positive effect on the surface roughness and microstructure observations. Moreover, the small value of the step size has a positive effect on both the maximum forming angle and the surface quality for Al/SUS and SUS/Al bimetal sheets.
4. The best structure is observed in the case of the SUS layer forming the internal surface in the SUS/Al bimetal sheet using a large tool diameter and small step size. Furthermore, the roughening in the case of the Al layer forms the external surface is higher than that in the case of the SUS layer, especially with a small tool diameter.
5. The microstructure observations demonstrated that the contact surfaces experience micro cracks and striations while the non-contact surfaces experience orange peel and micro voids which are responsible for the roughening of the Al/SUS bimetal sheets.
6. The effect of layer arrangement on the surface roughness is higher than that on the maximum forming angle. The higher maximum forming angle with better surface quality is found to be for the SUS/Al layer arrangement rather than the Al/SUS layer arrangement.

## Figures and Tables

**Table 1:** Numeric and categorical factor levels.

Parameter	Unit		Levels	
		1	2	3
Tool diameter	mm	10	15	20
Feed rate	mm/min	1000	2000	3000
Step size	mm	0.15	0.57	1
Layer arrangement	-	Al/SUS	SUS/Al	-

**Table 2:** Design matrix and experimental results.

Test	Numeric Factors			Categorical Factor	Responses	
	d (mm)	f (mm/min)	∆z (mm)	(LA)	θ (°)	Ra (μm)
1	10	3000	1	SUS/Al	57.59	2.947
2	15	1000	0.15	Al/SUS	63.22	0.783
3	20	2000	0.57	Al/SUS	66.38	0.694
4	10	1000	1	Al/SUS	60.85	2.552
5	20	2000	0.57	SUS/Al	64.93	0.682
6	15	1000	1	SUS/Al	63.77	1.678
7	20	1000	1	Al/SUS	65.88	1.091
8	10	3000	0.15	Al/SUS	64.94	0.986
9	10	1000	0.57	SUS/Al	68.75	1.443
10	10	2000	0.15	SUS/Al	66.95	1.934
11	20	1000	1	Al/SUS	65.35	1.045
12	15	3000	0.15	SUS/Al	63.48	1.244
13	20	3000	1	SUS/Al	65.54	0.735
14	10	2000	0.15	SUS/Al	66.13	1.825
15	10	3000	0.15	Al/SUS	65.12	1.173
16	15	2000	1	SUS/Al	61.66	2.167
17	15	1000	0.15	SUS/Al	67.16	1.134
18	20	3000	0.15	Al/SUS	64.39	0.845
19	15	3000	1	Al/SUS	64.68	2.187
20	10	2000	0.57	Al/SUS	64.10	1.648
21	10	1000	0.57	SUS/Al	67.81	1.484
22	20	1000	0.15	SUS/Al	63.83	0.512

**Table 3:** Optimum parameters of the predictive models.

Response	M	H	Lr	MAPE (%)
θ	3000	3	0.01	1.161
Ra	2000	3	0.01	6.865
